# Prospective Comparative Study Between Proximal Femoral Nail vs. Screw Augmented Proximal Femoral Nail in Unstable Intertrochanteric Fractures of Femur

**DOI:** 10.7759/cureus.32791

**Published:** 2022-12-21

**Authors:** Ajay K Rajput, Pradeep K Gupta, Simrat Pal S Gill, Santosh Kumar Singh, Manish Raj, Jasveer Singh, Pawan Dubey, Pranav Sharma

**Affiliations:** 1 Department of Orthopaedics, Uttar Pradesh University of Medical Sciences (UPUMS), Etawah, IND; 2 Department of Orthopaedics and Traumatology, Employee's State Insurance Corporation (ESIC) Hospital, Jajmau, Kanpur, IND; 3 Department of Orthopaedics, All India Institute of Medical Sciences, Deoghar, Deoghar, IND

**Keywords:** pfn, screw-augmented, proximal femoral nail, unstable, trochanteric fractures, lateral wall augmentation, intertrochanteric

## Abstract

Introduction

The proximal femoral nail (PFN) is a widely accepted fixation method for the management of unstable intertrochanteric fractures. Reconstructing the lateral trochanteric wall and ensuring the stability of the trochanteric fragments are considered to be essential for enhancing the prognosis of unstable intertrochanteric fractures. The aim of this study is to evaluate and compare the results of the management of unstable intertrochanteric fracture of the femur using PFN and the screw-augmented PFN (aPFN).

Methods

This prospective comparative study was undertaken from January 2020 to July 2021 and included 60 patients presenting with unstable intertrochanteric fractures (AO/OTA type 31-A2.2 and 31-A2.3) at a tertiary care teaching institute in northern India. The enrolled patients were randomly divided into two groups (group 1 and group 2) and were managed with screw-augmented PFN and PFN, respectively. Functional outcome was evaluated using the Salvati and Wilson score at the 12-month follow-up. SPSS version 24.0 (IBM Corp., Armonk, NY, USA) was used for statistical analysis. A p-value less than 0.05 was regarded as significant.

Results

The average time to union of the fractures in group 1 was 12.66 ± 1.68 weeks, while it was 13.47 ± 1.47 weeks in group 2 (p = 0.055). At the 12-month follow-up, the average functional outcome, as evaluated by Salvati and Wilson score, was 34 ± 2.40 in group 1, whereas it was 31.58 ± 4.4 in group 2; and the difference was observed to be statistically significant (p = 0.011). Group 1 had 28 patients (93.33%) with excellent to good results, while group 2 had 25 patients (83.33%) with excellent to good results. One patient in group 1 and five patients in group 2 had poor outcomes at the 12-month follow-up.

Conclusion

Screw-augmented PFN has better functional outcomes as compared to PFN alone for the management of unstable intertrochanteric fractures. Hence, in our opinion, screw augmentation of PFN may be the better fixation technique for most unstable intertrochanteric femur fractures.

## Introduction

Intertrochanteric fractures are the most common proximal femoral fractures, which occur in the region extending from the extracapsular basilar neck to the region along the lesser trochanter, proximal to the starting of the medullary canal. These are more commonly seen in geriatric patients. They are three to four times more common in females with osteoporosis, with trivial falls being the most common mechanism of injury [1]. As the general life expectancy has increased in the past two decades, the incidence of proximal femur fractures is also increasing, leading to increased mortality and morbidity, particularly in the younger age group [2]. Even minor trauma is enough to cause proximal femur fractures in geriatric patients due to pre-existing osteoporosis, decreased muscle power, decreased reflexes, poor vision, and labile blood pressure, whereas in younger patients it requires high-energy trauma [3].

In 1990, of all hip fractures, 26% occurred in Asia. This is expected to rise to 37% in 2025 and 45% in 2050 [3]. In the elderly, 50% of fractures around the hip are trochanteric fractures, and 35-40% of these are unstable intertrochanteric fractures [4].

Based on the fracture patterns, these fractures are typically divided into stable and unstable fractures. Undisplaced fractures and fractures with intact posteromedial cortex are classified as stable, whereas fractures with posteromedial comminution, loss of lateral wall, reverse obliquity type, and fractures with four or more parts are classified as unstable fractures, which account for approximately 50%-60% of all intertrochanteric fractures [5,6]. Surgical management by internal fixation of these fractures increases patient comfort, facilitates nursing care, decreases hospitalization, and reduces complications of prolonged recumbency [7].

Unstable inter-trochanteric fractures of the femur (AO 31A2, AO 31A3) [8] continue to be a challenge for orthopedic surgeons because, despite high union rates, the functional outcome still tends to be disappointing [9,10]. An intact lateral wall plays a key role in the stabilization of unstable intertrochanteric fractures by providing a lateral buttress for the proximal fragment, and its deficiency leads to excessive collapse and varus malpositioning [6,11].

Intramedullary nails, sliding plate devices alone or in combination with trochanteric stabilization plates, proximal femoral locking plates, and angular blade plates are the options for managing unstable trochanteric fractures [12]. Sliding plate devices, whenever used for unstable intertrochanteric fractures with lateral wall comminution, result in gross medialization of the distal fragment with an excessive collapse of the proximal fragment, leading to implant failure with failure rates up to 50% [13]. Intramedullary devices like the proximal femoral nail (PFN) have been reported to have an advantage in such fractures as their placement allows the implant to lie closer to the mechanical axis of the extremity, thereby decreasing the lever arm and bending moment on the implant [14]. The intramedullary position of PFN and it being a load-sharing device prevents the medialization of the distal fragment and excessive collapse of the proximal fragment, with less operative time and blood loss, and allows early weight-bearing with less resultant shortening on long-term follow-up [15,16].

Various studies have shown that an intact lateral trochanteric wall gives the proximal fragment biomechanical support and a lateral buttress, making it more stable, while the fractured lateral trochanteric wall makes it more unstable, leading to shaft medialization, varus fixation, and fracture collapse [17-19]. So the integrity of the lateral trochanteric wall is absolutely necessary for proper fracture fixation. An increase in the stability of fixation can be achieved when the lateral wall is augmented with a screw, a cerclage wire, or a plate [20]. Intrinsic factors like osteoporosis and comminution of the fracture, and extrinsic factors like the choice of reduction technique, choice of implant, and technique of insertion affect the outcome of internal fixation.

Therefore, to verify whether there are any differences regarding the clinical and functional outcomes, and the adequacy of and the time taken for radiographic union between PFN and screw-augmented PFN (aPFN) in the management of unstable intertrochanteric femur fractures (AO/OTA type 31-A2.2 and 31-A2.3), this comparative study was undertaken to prospectively weigh the results with both the fixation methods for any significant differences.

## Materials and methods

Objective

This study was conducted with the objective of evaluating and comparing the outcomes of the management of unstable intertrochanteric fractures of the femur using PFN and screw-augmented PFN (aPFN) on the basis of the Salvati and Wilson score [21].

Study design and area

A hospital-based prospective comparative study was conducted at the department of Orthopaedics of a tertiary care hospital in Etawah district of the central region of the state of Uttar Pradesh, India.

Study period

This study was carried out from January 2020 to July 2021, including a minimum follow-up of 12 months.

Study population

All the patients admitted to the department of Orthopaedics at a tertiary care hospital in the central part of the state of Uttar Pradesh, India, with the diagnosis of unstable intertrochanteric fractures were screened as per the inclusion and exclusion criteria and subsequently enrolled and managed with screw-augmented proximal femoral nailing (Group 1) and proximal femoral nailing alone (Group 2), and followed-up for a minimum of 12 months between January 2020 and July 2021. Those who completed the follow-up duration were included in the final study population. Institutional Ethical Committee approval was taken prior to the start of the study (ethical clearance reference number- 1709/UPUMS/Dean/M/Ethical/130/2019-20, Institutional Ethical Committee, Uttar Pradesh University of Medical Sciences). The following inclusion and exclusion criteria were used for the selection of patients in the study:

Inclusion Criteria

1. Patients above 18 years of age, 2. Unstable inter-trochanteric fracture (AO 31 A2, AO 31 A3 as per AO/OTA classification), 3. Able to walk prior to sustaining the fracture.

Exclusion Criteria

1. Presence of active infection, 2. Patients medically unfit for surgery, 3. Patients with chronic illnesses and immunocompromised conditions, 4. Patients not willing to participate in the study and who did not complete the follow-up for a minimum of 12 months, 5. Open fractures and pathological fractures.

Sample size

**Figure 1:**
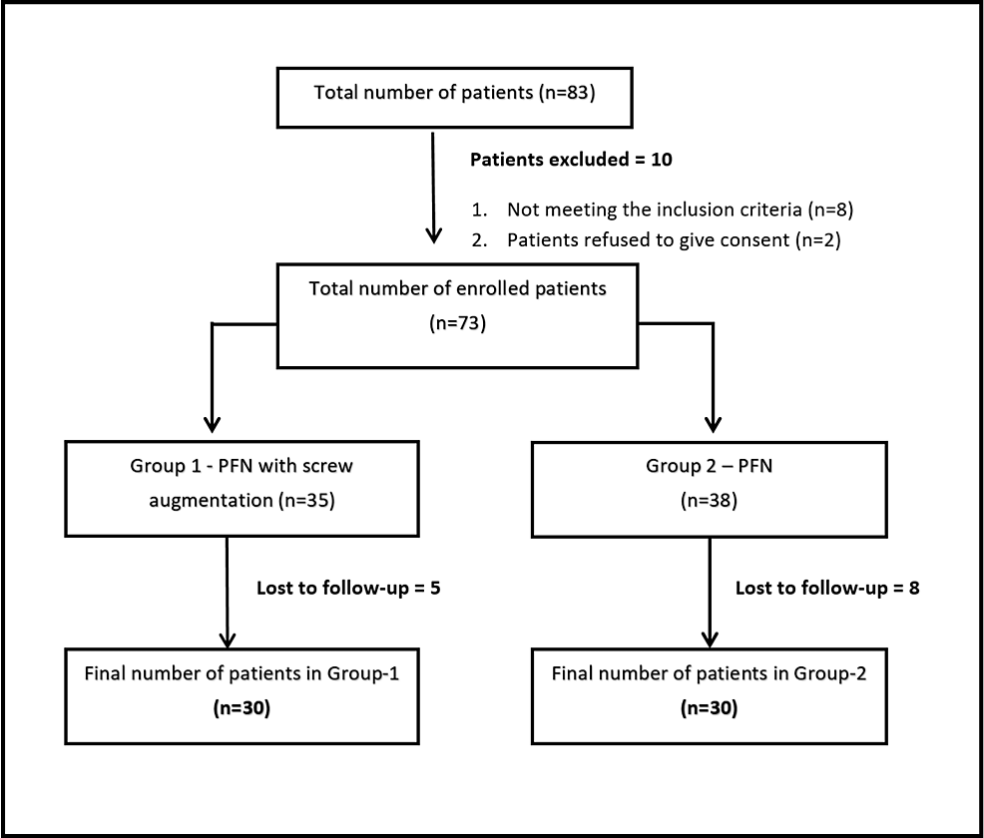
Flowchart depicting the methodology adopted for patient enrollment PFN: Proximal femoral nail

This prospective comparative interventional study involved 60 patients with unstable intertrochanteric fractures of the femur admitted to the department of Orthopaedics and subsequently managed with either of the two fixation methods, with a completed 12-month follow-up duration during the study period (January 2020 to July 2021). The purposive method of sampling was used to collect the sample population. The patients were randomly divided into two groups of 30 each (group 1 and group 2) using a computer-generated table to minimize the selection bias and were managed with screw-augmented PFN and PFN alone, respectively. Informed consent was obtained from all the patients before their enrollment in the study. The methodology adopted for the selection of the study population is depicted in Figure 1.

Preoperative evaluation

Detailed history and thorough clinical examination, sensory-motor examination, evaluation of the limb vascularity, deformities of the hip, the status of other joints, the ambulatory status of the patient before the injury, and any focus of infection in the body were conducted. All routine preoperative investigations were performed which included a complete blood count, blood group, random blood sugar, kidney function tests, liver function tests, serum electrolytes, CRP levels, Viral markers (for hepatitis B and C, and human immunodeficiency virus infections) and an electrocardiogram. Standard digital radiographs of the pelvis with bilateral hips in 15° internal rotation and an anteroposterior and lateral view of the affected hip with thigh were taken of all the patients. Classification of the fracture was done as per AO/OTA classification.

Surgical procedure

All the patients were operated on by a team of three experienced surgeons. In Group I patients, closed reduction and internal fixation of the fractures were done using PFN augmented with a screw in the greater trochanter area. In Group 2 patients, closed reduction and internal fixation by PFN alone were done.

The patients were placed in the supine position on an orthopaedic fracture table under spinal anesthesia. The unaffected lower limb was flexed and abducted to allow uninterrupted and easy access for the image intensifier. Under an image intensifier, the fracture was closely reduced by traction with slight internal or external rotation depending on the nature of the fracture, followed by the provisional fixation of the fracture segments with the help of 3 mm K-wires anteriorly. The purpose of reduction was to provide weight-bearing stability as well as address varus and rotational deformities. A 3-5 cm lateral skin incision was made extending from the tip of the trochanter and spanning proximally, followed by the gluteus-medius aponeurosis being split along its fibers, and subsequently, the gluteus-maximus aponeurosis being split. The entry point was made with a curved bone awl just medial to the tip of the greater trochanter. A guide wire was passed through the entry site into the femoral canal under image guidance. Reaming was done by successively increasing sized reamers, followed by the proximal femoral reamer to ream the proximal portion of the femur. In this study, a 9, 10, or 11-mm nail was used, depending on the diameter of the femoral canal. The 6° mediolateral angle nail was then mounted to the jig and passed over the guide wire into the proximal femur, crossing the fracture site into the femoral shaft. After the insertion of PFN, two 3.2-mm threaded tip guide wires were inserted beyond the fracture site into the femoral head under the guidance of the image intensifier, such that their directions were parallel to the neck and above the calcar. The head and neck of the femur were reamed under radiographic guidance for the cannulated 8-mm hip screw (70 to 110 mm in length) and the cannulated 6.4-mm stabilization screw (60 to 100 mm in length). The hip screw was inserted into the lower half of the neck of the femur within a 5-10 mm distance from the subchondral bone of the femoral head, and the stabilization screw was inserted into the proximal slot of the nail. Both screws were tightened alternately to secure the proximal fixation. Then the guide wires and the K-wires were removed, and distal locking bolts were tightened into the nail via the jig by the small stab incisions over the lateral thigh.

Screw augmentation

For screw augmentation of the PFN, a guide wire was inserted under the image intensifier guidance at the anterolateral area of the greater trochanter, up to the inferior part of the femoral head using a small incision. Drilling was done over the guide wire using a cannulated drill bit. A corticocancellous screw of 6.5 mm was passed over the guide wire for augmentation. A screw of appropriate length was inserted and tightened. All the incisions were closed and sterile dressings were applied.

Postoperative care protocol

All patients were given intravenous antibiotics and deep venous thrombosis prophylaxis according to the standard hospital protocol. Patients were allowed to sit up in bed on the second postoperative day. Static quadriceps exercises were started on the second or third postoperative day after adequate pain relief. Sutures were removed on the fourteenth postoperative day and the patients were discharged. Patients were mobilized with non-weight bearing as soon as the pain or general condition permitted. Weight-bearing was commenced depending upon the stability of the fracture and adequacy of fixation, delaying it for patients with unstable fixation.

Follow-up

The patients were followed up postoperatively at one, three, six, and 12 months duration. Serial radiographs were taken to assess for fracture union and for complications like nonunion, malunion, screw cut-out, implant failure, and avascular necrosis of the femoral head. The fracture was said to have united when there was an absence of pain at the fracture site clinically on palpation and an appearance of a radiographic bridging callus with a minimum continuity of three cortices on anteroposterior and lateral radiographs. The fracture union was considered as malunion if varus angulation was greater than 10 degrees.

Functional outcome and statistical analysis

Functional outcome was assessed according to the Salvati and Wilson score which includes pain, function, muscle power-motion, and walking ability [21]. A total score of less than 16 was considered poor, a score of 16-23 fair, a score of 24-31 good, and a score of more than 31 was considered excellent. Microsoft Excel was used to compile the data, and IBM SPSS Statistics for Windows, Version 24.0 (IBM Corp., Armonk, NY, USA) was used for data analysis. Tables were created using frequency, percentage, mean, and standard deviation. The relationship between the data was determined using the Chi-square test (Χ^2^), and the Student t-test. A p-value less than 0.05 was regarded as statistically significant.

## Results

Sixty patients with unstable intertrochanteric fracture (AO/OTA type 31-A2.2 and 31-A2.3) fulfilling the inclusion and exclusion criteria were included in the study and were randomly distributed in Group 1 and Group 2 (30 patients in each group) treated with aPFN and PFN alone, respectively. All the demographic characteristics and intraoperative variables of the patients are depicted in Table 1.

**Table 1:** Demographic and intraoperative variables. ^1^The data is presented in Mean ± Standard deviation (SD) RTA: Road traffic accident, PFN: Proximal femoral nail.

S.N.	Variables		Group-1 (Screw augmented PFN group, N)	Group-2 (PFN group, N)	p-value
1	Age (years)^1^		59.03 ± 16.10	60.67 ± 13.21	0.99
2	Sex, male/female		18/12	22/8	0.27
3	Side involved, right/left		15/15	13/17	0.60
4	Mode of injury	Fall from height	3	5	1.00
		RTA	6	5	
		Slip on ground	21	20	
5	Fracture classification (AO/OTA type)	31A2	17	20	0.43
		31A3	13	10	
6	Injury surgery interval (days)^1^		8.03 ± 1.35	8.15 ± 1.74	0.77
7	Duration of surgery (minutes)^1^		68.67 ± 8.99	67.17 ± 5.826	0.44
8	Intraoperative blood loss (ml)^1^		104 ± 14.32	103 ± 13.17	0.64
9	Fluoroscopy time (number)^1^		46.81 ± 3.1	45.37 ± 1.2	0.02

The mean age in group 1 was 59.03 ± 16.10 (range 41 to 85 years), with a male-to-female ratio of 3:2 and a left-to-right ratio of 1:1. Twenty-one patients sustained injuries due to slip and fall on the ground, six patients sustained injuries due to road traffic accident (RTA), and three patients sustained injuries due to falling from height.

In group 1, 17 patients had 31A2 type and 13 patients had 31A3 type fracture patterns while in group 2 twenty patients had 31A2 type and 10 patients had 31A3 type fracture patterns. In group 1, the average injury to surgery duration was 8.03 ± 1.5 days while it was 7.06 ± 1.74 days in group 2. The mean duration of surgery in group 1 was 68.67 ± 8.99 minutes, whereas it was 67.17 ± 5.82 minutes in group 2 (p = 0.44). The estimated mean blood loss in group 1 was 104 ± 14.32 ml, while in group 2, it was 103 ± 13.17 ml, but no significant difference was observed (p = 0.64). None of the patients received an intraoperative blood transfusion.

The fluoroscopy time used during the surgery was estimated as an average number of c-arm shots used during the operation. In group 1, the number of shots used (46.81 ± 3.1) were significantly more (p = 0.021) than in group 2 (45.37 ± 1.2).

All the postoperative characteristics of the patients are depicted in Table 2.

**Table 2:** Post-operative variables ^1^The data is presented in Mean ± Standard deviation (SD).

S.N.	Post-operative variables		Group-1 (Screw-augmented PFN group, N)	Group-2 (PFN group, N)	p-value
1	Post-operative blood transfusion required		6	5	0.74
2	Time of fracture union (weeks)^1^		12.66±1.68	13.47±1.47	0.055
3	Functional Outcome score (Salvati and Wilson functional Score)^1^	at 3 months	20.8±3.5	19.06±2.8	0.038
		at 6 months	29.26±4.80	25.93±4.4	0.006
		at 12 months	34±2.40	31.58±4.4	0.011
4	Limb length shorting (mm) at final follow-up^1^		10.71±1.59	13.68±1.37	0.01
5	Revision surgery by arthroplasty		1	5	0.085
6	Complications	Infection	1	1	0.015
		Screw back out/impingement	1	5	
		Z-effect/reverse-Z effect	1	3	
		Varus malunion	1	4	
		Interlocking screw breakage	1	1	
		Anterior thigh pain	1	1	

Patients with hemoglobin levels less than 10 g/dL postoperatively received blood transfusions according to the standard hospital protocol. Consequently, six patients in group 1 and five patients in group 2 received blood transfusions (p = 0.74).

The average union time of the fracture in group 1 was 12.66 ± 1.68 weeks, while it was 13.47 ± 1.47 weeks in group 2 (p = 0.055).

At the 12-month follow-up, the average functional outcome was evaluated by Salvati and Wilson score, in group 1 the score was 34 ± 2.40, whereas it was 31.58 ± 4.4 in group 2, and the difference was found to be statistically significant (p = 0.011). Group 1 had 28 patients (93.33%) with excellent to good results, while group 2 had 25 patients (83.33%) with excellent to good results. One patient in group 1 and five patients in group 2 had poor outcomes at the 12-month follow-up.

Superficial infections were seen in one patient in each group. It was managed with dressings and oral antibiotics as per culture-sensitivity reports.

Only one of the patients from group 1 encountered the complication of screw backing out of the proximal femur during the 12-month follow-up, but the patient had no clinical symptoms and did not need revision surgery. Five patients in group 2 experienced femoral head screw backout and lateral thigh pain and limp, requiring a revision with a screw exchange, however, all of them achieved fracture union by the completion of one-year follow-up and had good to excellent scores.

The femoral neck-shaft angle was evaluated at the final 12-month follow-up, and a varus deformity was observed in one of the patients in group 1, who had no clinical symptoms. In group 2, four patients reported varus deformity, out of which two patients experienced minor pain in the hip joint while walking and had a fair hip score, while the other two patients complained of severe pain at the hip joint while walking, which required revision surgery in the form of hip arthroplasty. One patient in either group reported breakage of the distal interlocking bolt, which was managed by an exchange of the bolt. One patient in either group complained of anterior thigh pain, but it was not clinically significant, and no revision was required for them. Revision surgery by total hip arthroplasty was also required for three patients from the non-augmented group (group 2) due to head screws migrating proximally through the femoral head. One patient in the augmented group (group 1) reported screw penetration and required revision arthroplasty surgery. The rate of revision surgeries by the arthroplasty was nonsignificant between the two groups (p = 0.085).

The complication rates were found to be significantly higher in group 2 as compared to the patients in group 1 (p = 0.015). No case of nonunion was observed in either of the groups.

**Figure 2:**
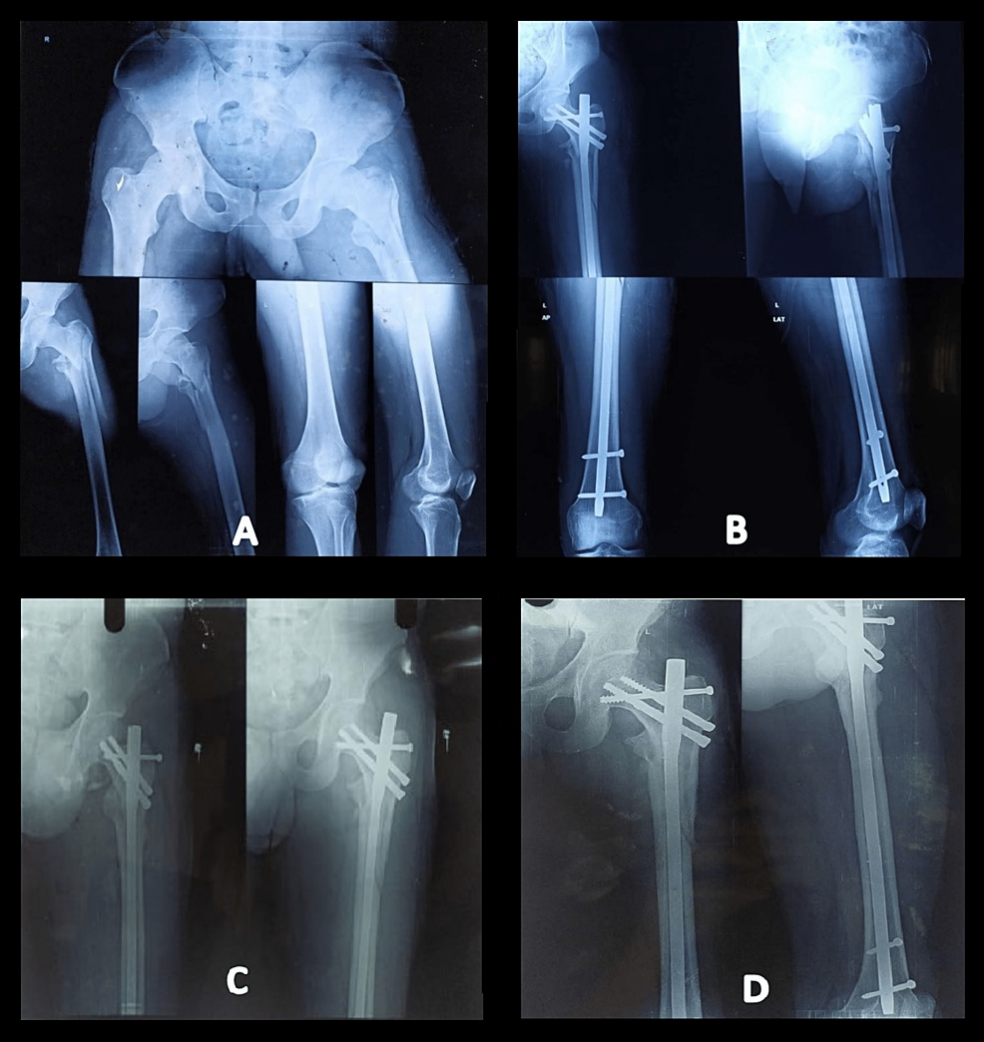
Serial follow-up radiographs of a 31-year-old male with unstable intertrochanteric fracture (AO/OTA type 31-A2.3) of the left side managed by proximal femoral nail augmented with screw A: Pre-operative radiographs showing the sustained fracture pattern B: Immediate postoperative radiograph of the fracture managed with PFN with augmentation screw C: Follow-up radiograph three months after surgery D: Follow-up radiograph 12 months after surgery

The serial radiographs of a case of a 31-year-old male who sustained a post-traumatic unstable intertrochanteric fracture (AO/OTA type 31-A2.3) of the left side which was managed with a proximal femoral nail augmented by a screw are demonstrated in Figure 2.

**Figure 3:**
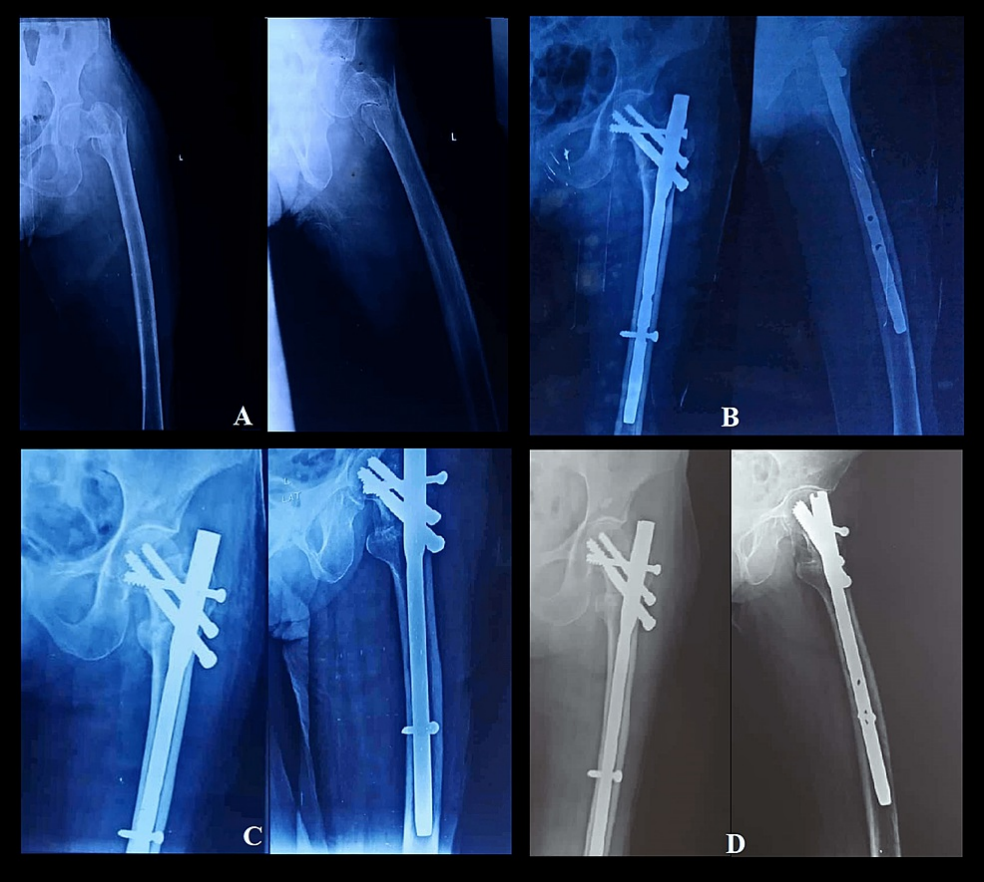
Serial follow-up radiographs of left side intertrochanteric fracture femur (AO/OTA 31-A2.2) in a 67-year-old female managed with proximal femoral nail and augmentation screw A: Preoperative radiographs of the sustained fracture B: Postoperative radiographs immediately following the surgery C: Radiographs at three-month follow-up D: Radiographs at the final follow-up 12 months postoperatively

The sequential follow-up radiographs of a 67-year-old female with an unstable intertrochanteric fracture (AO/OTA type 31-A2.2) of the left side managed with a proximal femoral nail and an augmentation screw are depicted in Figure 3.

## Discussion

The goal of this study was to compare the functional outcome of patients with unstable intertrochanteric fractures treated with two different fixation devices, the proximal femoral nail with screw augmentation and the proximal femoral nail alone. Different types of intramedullary and extramedullary devices are present for the fixation of these types of fractures, however, intramedullary fixation using PFN is gaining preference over sliding hip screw devices due to numerous advantages, namely, less shortening of the limb, less collapse at the fracture site, and less implant failure rates.

Al-yassari et al. [22] conducted a prospective study comprising 76 patients with the diagnosis of unstable intertrochanteric fractures. They reported that the outcome according to the Salvati and Wilson hip function scoring system was either good or very good in 94% of the patients and the level of function was similar to the pre-injury level in 50% of the patients. They also concluded that the PFN was a useful device in the treatment of unstable trochanteric femoral fractures and it was a relatively easy procedure and a biomechanically stable construct allowing early weight bearing. A morphological analysis conducted by Wei et al. [23], which included 46 patients with unstable intertrochanteric fractures (AO/OTA 31-A1 and AO/OTA 31-A2) managed by intramedullary nailing, reported complete osseous healing of the fractures without any complication such as nonunion and loosening of internal fixation.

The traditional description of the posteromedial fracture part as the most important prognostic factor has been revised because recent studies have insisted that the lateral wall fracture also constitutes a type of unstable fracture and that the integrity of the lateral wall is clearly indicated for event-free fracture healing and that the fracture of the lateral wall should be avoided in any fixation treatment [17]. Fan et al. [24] conducted a retrospective study that included 130 intertrochanteric fractures with lateral femoral wall fractures treated with intramedullary fixation and they reported that the comminution extent of the lateral femoral wall fracture might influence the stability of intertrochanteric fractures, and suggested the use of intramedullary fixation might be an effective treatment method. Jain et al. [25] reported in their study that the lateral trochanteric wall in intertrochanteric fractures is significantly important, and when the lateral wall is broken, it can lead to poorer results, and in such cases, the augmentation of PFN with a trochanteric buttress plate provides faster union, early weight bearing, better reduction, and hence better hip functions but at the cost of surgical time, blood loss, and radiation exposure.

In the present study, we observed that at 12 months postoperatively, the average Salvati and Wilson score for the augmented PFN group was 34 ± 2.40 whereas it was 31.58 ± 4.4 for the PFN group, which was found to be a statistically significant difference (p = 0.011). Group 1 had 28 patients with excellent to good results (93.33%) while group 2 had 25 (83.33%) such patients. One group 1 patient and five group 2 patients had unsatisfactory 12-month results.

In the augmented PFN group, one patient had a varus deformity but showed no clinical symptoms, whereas in group 2, four patients had the deformity and two of them had severe hip pain that necessitated revision surgery by arthroplasty. Three patients in the non-augmented group (group 2) needed revision surgery by total hip arthroplasty as a result of the head screws penetrating the femoral head. One patient in the augmented group (group 1) experienced screw penetration and needed arthroplasty surgery for revision. Between the two groups, the frequency of revision arthroplasty procedures was not statistically significant (p = 0.085). Patients in group 2 had a considerably higher complication rate than those in group 1 (p = 0.015). No instance of nonunion was noticed in any of the groups. The results of our study are comparable to the prospective study conducted by Gadegone et al. [26] in which patients with unstable trochanteric femoral fractures were fixed by PFN with augmentation by an additional screw from the trochanter to the inferior quadrant of the femoral head or cerclage wire to strengthen the lateral trochanteric wall. They reported that bone healing was observed in all cases over a 14.2-week period. They observed complications in nine patients including lateral migration of neck screws, the Z-effect, infection, and distal interlocking bolt failure. At the final follow-up in their study, the Salvati and Wilson hip function was 32 (out of 40) in 88% of the patients, and they concluded that stabilizing the lateral trochanteric wall with additional screws or cerclage wire improves the construct's stability. In another such study on unstable intertrochanteric fractures by Huang and Wu [27], they concluded that the use of cerclage cable for better fracture stabilization along with PFN led to superior outcomes in terms of Harris Hip Score, time for fracture healing and weight-bearing, with reduced incidence of postoperative complications and improved self-care by the patients.

In their study, Kulkarni et al. [20] concluded that the use of cerclage wires and lag screws for lateral wall reconstruction to augment the fixation of the intramedullary nail in unstable trochanteric fractures has been shown to be effective in decreasing complications and achieving a favorable radiological and functional outcome. There is minimal blood loss and soft tissue injury, and very little extra time is needed for the operation. Purushotham et al. [28] conducted a prospective study and observed that in unstable intertrochanteric fractures, enhancing the proximal femoral nail with an extra screw or cerclage wire improves the efficacy and stability of the construct, which in turn facilitates union and shortens the time to union.

Although PFN with screw augmentation has slightly more blood loss, a slightly longer duration of surgery, and more radiation exposure as compared to PFN, screw-augmented PFN is better in terms of functional outcome. Although we did not find any significant difference in fracture union time between the two groups, the patients managed with screw-augmented PFN had a better functional hip outcome, lesser postoperative limb shortening, and fewer implant-related complications like screw back-out and screw breakage, as compared to patients managed with PFN alone. This change is most likely attributed to the lateral buttressing effect of screw augmentation.

Limitations

Although our study showed that there is a significant advantage of screw augmentation of PFN in the management of unstable intertrochanteric fractures of the femur, some limitations of our study that need addressing are a small sample size, short-term follow-up, and an unknown biomechanical principle of augmentation of the PFN. Thus further biomechanical and clinical studies are necessary to evaluate the efficacy of PFN augmentation.

## Conclusions

In the present study, it has been observed that the use of an augmentation screw along with PFN in the management of unstable intertrochanteric fractures, particularly with lateral wall fractures, resulted in significantly improved functional outcomes along with the advantages of lesser postoperative complications and fewer revision surgeries. Therefore, we conclude that PFN with screw augmentation has overall better results as compared to PFN alone. Hence, in our opinion, screw augmentation of PFN may prove to be a better fixation technique for most unstable intertrochanteric femoral fractures.
